# The medical art towards new paradigms

**DOI:** 10.1007/s40620-024-01982-z

**Published:** 2024-06-26

**Authors:** Ortensio Zecchino

**Affiliations:** Biogem, Molecular Biology and Genetics Institute, Via Camporeale snc, 83031 Ariano Irpino, AV Italy

Medicine and health are inextricably linked to life, between the alpha of the labor of birth (Leopardi's verses remind us of this: "*Man is born* in labor: and there's a *risk of death* in being *born*. ") and the omega of death.

Medicine is a field from which no human is a stranger. Foucault wrote: *Hence the decisive place of medicine in the overall architecture of the human sciences* [1] (210).

Yet difficulty in defining the essence of medicine arises precisely from the fact that it is closely connected to human life.

Literature, which is a mirror of life, provides a rich array of writings about physicians and medicine.

One of the most vivid descriptions of the doctor-patient relationship is on the first page of Marguerite Yourcenar’s *Memoirs of Hadrian*. Hermogenes, Hadrian's physician—shows sincere ‘compassion’ and tries to downplay the severity of the disease— while the emperor has an unprecedented feeling of helplessness and declares he feels like a simple aggregation of flesh and blood, stripped not only of the dignity of an emperor, but even of that of a man.

In this page, even beyond words, we can feel the emotions that animate the complex and difficult doctor-patient relationship. There is the anxious wait for the verdict, the trust in the doctor, the awareness of the limits of science, and an implicit invitation to avoid therapeutic obstinacy. But above all, the patient’s feeling of smallness and impotence in front of the doctor emerges, with universal relevance.

All these feelings are the breeding ground for the patient’s mistrust and not uncommon dull hostility towards physicians, arising from an unacknowledged disappointment for the unmet expectations of salvation or from a sort of envy towards the doctor’s dominant position compared to the patient’s weakness.

Petrarch’s well-known iatrophobia arose precisely from this feeling.

Nowadays, the erroneous idea that medical progress must inevitably correspond to a positive outcome is increasing the patient’s and family’s disappointment when results do not meet expectations, and the idea that the physician is responsible for the failure is increasingly widespread. This leads to physical violence against doctors, carried out in contexts where people take justice into their own hands; this also leads to the growing inclination to seek justice in court, assuming professional negligence a priori. This has negative consequences, including the decreasing number of physicians attending some specialization schools and the increasing number of doctors leaving front-line departments.

Among authors who have dedicated critical attention to medicine, Michel de Montaigne stands out as a writer who focused on the fundamentals of medicine, highlighting them with his sharp writing.

From the belief that medicine is based on an experiential approach, and that experience, however rich and varied it may be, often proves powerless compared to the absolute singularity of diseases, which is a mirror of the absolute singularity of each patient, Montaigne supports that, ultimately, chance is the master of medical choices. Accordingly he writes:

The doctor is presented with many diseases and circumstances, but experience does not allow getting predictable results, because human wisdom does not allow orientation.

*[…]. When recovery then has occurred, how can one be sure that it was not because the disease had run its course, or an effect of the case, or the influence of something else eaten or touched that day, or the efficacy of his grandmother’s prayers? And how many times would it be necessary to reconstruct the long chain of cases and coincidences to infer a rule from it? And what will happen if someone else has had opposite experiences* [2]*?* (pp. 1447–1449).

With these reflections, the skeptic Montaigne touched the core of medicine, grounded on cases and probabilism, which can have, and indeed do have exceptions.

Similar thoughts had already been expressed two millennia ago by Aulus Cornelius Celsus:

*Being Medicine a conjectural art and being specific of conjecture that while it often aligns with reality, it sometimes fails*. [3] (p. 63).

Along this line, literature has flourished on the phenomenon of spontaneous oncologic recoveries, which contradicts consolidated larger scale findings.

Presently, with the succession of scientific revolutions, the empirical approach of medicine has evolved into increasingly scientific terms. Before our eyes we see a great deal of extraordinary progress. Let us cite the diagnostic imaging and endoscopic techniques that allow the miracle of seeing into the hidden recesses of our organs, and the new insights revealed by the study of the microbiota.

Another innovation, that can look almost like science fiction, is the generation of organoids. Until now, to test drugs we used animal models, mainly mice, which have a genome that is approximately 95% similar to ours. Nowadays, starting from patients’ cells, it is possible to replicate a specific organ in 3D, upon which testing the effectiveness of existing drugs or, depending on their characteristics, producing ad hoc drugs or vaccines.

Yet some great revolutions are overturning the foundations of research methods and therapeutic approaches.

The first is the genetic revolution that, after a 50-year journey that began in the early twentieth century and culminated in the discovery of the double helix, ultimately led to the sequencing of the entire human genome. Thanks to structural genomics, we now know the number and type of genes that make up the human body, even if we do not yet know all their specific functions, and especially, what the characteristics and the effects of their interaction are, which is the specific topic of functional genomics. In the last ten years, medical genetics has made it possible to identify over a thousand new diseases and hundreds of disease genes [4] (p. 374).

Another important revolution is the so-called omics sciences, capable of providing extensive information even on biological systems.

A further unstoppable, rapidly evolving field is that of artificial intelligence with its limitless applications in the medical field.

All these extraordinary innovations, in turn, depend on the big data revolution, with the possibility of processing an astonishing quantity of data.

Yet, if for some time we had the illusion that genetics could establish clear cause-and-effect laws, epigenetics made the dream of deterministic rigor vanish, opening new horizons [5] (391).

In this regard, we could recall what has been even more shocking in the physics of the last century which has dismantled the traditional deterministic approach to affirm the quantum-probabilistic paradigm.

Looking back, we can say that epigenetics has given scientific dignity to an intuition already present in the Hippocratic teaching.

In a text of the *Corpus Hippocraticum*, entitled *On Ancient Medicine*, it is written: *It is absolutely necessary for every medical doctor to study the human nature; if he wants to fulfill his own duty, he needs to know the relationships between man and his food, his beverages, and with his overall life style, and the influence that each element has on the others* [6]*.* (p. 14).

All this allows delving ever deeper into the condition of the individual patient, so that the disease as an abstract category is no longer at the center of medicine, increasingly replaced by the patient in his/her singularity.

This shows that Montaigne had grasped the focus of the problem. Of course he was exaggerating, even if perhaps not too much at the time, in stating that, in the probabilistic approach, it was chance that most often played the main role in medicine.

The combination of these revolutions has led to “personalized medicine” (in the American context, it seems that “precision medicine” is preferred, even if it is possible to identify subtle differences between the two expressions).

Personalized medicine is a new way of understanding age-old medical practice: prevention and treatment are grounded in the identification of the genotypic characteristics of the individual patient as well as in the study of the patient’s specific lifestyle and his/her environment.

Indeed, all the above requires a hopefully generalized technological adaptation of healthcare facilities, and raises the issue of costs.

However, some risks are intrinsic to this new medicine.

In the last quarter of the last century, two iconic books were published: *The medical nemesis*, with the subtitle *The expropriation of health* by Ivan Illich and *The doctor in the age of technology* by Karl Jaspers [7, 8].

Both highlighted the danger of being overwhelmed by technological approaches. The dawn of the above-mentioned revolutions was barely visible, but both philosophers were able to anticipate advantages and risks deriving from the proclaimed overwhelming scientific and technological development.

All these risks are taking shape. Innovations once considered science fiction are a reality and risk transforming the physician into a piece of intricate machinery, making him/her a hyper-specialist, knowledgeable about particularities but ignorant about the entirety of human beings.

Making use of two expressions increasingly used in current medical language, *Evidence based medicine* (EBM) and *Narrative based medicine* (NBM), there is a risk that the direct doctor-patient relationship and the careful listening to the patient’s narrative, which play a central role in the second approach, may be sacrificed and like overshadowed by the ‘scientific’ evidence brought about by the extraordinary technology available today.

Once the ancient paradigms of medicine as an experiential discipline, based on case study abstraction, have been overcome, the risk is that the novelty of the emphasized personalized medicine translates into attention limited to specific parts of the patient’s body while the complex human originality disappears.

In the everlasting pursuit of the optimization of medical training, we hear calls to give space to the interpretative-dialogical nature of medical practice. This task was a part of ancient southern Italian Medical schools. An enlightened sovereign, Frederick II, in his famous Constitution of 1231, asserting his will to protect people from the incompetence of physicians, made it compulsory to carry out a very long training so as to make them not only experts in clinical and surgical practice, but also ready to listen to the patient and capable of making decisions with adequate critical scrutiny: for this reason, he ordered that medical students, before moving on to professional topics, had to study logic for three years.

This strict directive was aimed at enhancing well thought-out personal judgment regarding the measures to be adopted, as had already been stated over a thousand years earlier in Hippocrates’s oath.

Physician’s synthesis is fundamental in medical practice, and is informed by many elements: science, experience, listening, but also intuition (how many times have you heard praises of the doctor’s ‘intuition’?). This is why—medicine is not only a science but also an art.

In conclusion, the recent medical revolution can elevate the medical doctor to unimaginable heights, thanks to the power of scientific tools. However, these tools can overwhelm the physician, compressing and even erasing the ability to listen to the patient, making the physician an automaton without responsibility.

The balance and neutralization of these risks lie in the philosophical thought, focused on the human essence and on the mystery of life. This truth was expressed with special trepidation and dismay by Benedetto Croce, the day after the Hiroshima apocalypse of August 6, 1945:

*To ward off danger, and to draw from scientific discoveries the good that they can give, is required […] a greater advancement of the intellect, moral faith, religious spirit and, in a word, of the human soul*. [9]

There are three words attributed to Hippocrates and reported by Karl Jaspers in the above-mentioned book, which still convey the meaning and ethics of the medical profession: *iatros philosophos isòteos*, which can be interpreted as: when the medical doctor acts as a philosopher he/she acquires a divine power!
